# Maternal Arsenic Exposure and DNA Damage Biomarkers, and the Associations with Birth Outcomes in a General Population from Taiwan

**DOI:** 10.1371/journal.pone.0086398

**Published:** 2014-02-18

**Authors:** Wei-Chun Chou, Yu-The Chung, Hsiao-Yen Chen, Chien-Jen Wang, Tsung-Ho Ying, Chun-Yu Chuang, Ying-Chih Tseng, Shu-Li Wang

**Affiliations:** 1 Department of Biomedical Engineering and Environmental Sciences, National Tsing Hua University, Hsinchu, Taiwan; 2 Division of Environmental Health and Occupational Medicine, National Health Research Institutes, Zhunan, Miaoli County, Taiwan; 3 Department of Obstetrics and Gynecology, Chung Shan Medical University Hospital, Taichung, Taiwan; 4 Department of Obstetrics and Gynecology, Hsinchu Cathay General Hospital, Hsinchu, Taiwan; 5 Epidemiology Branch, National Institutes and Environmental Health Sciences, National Institutes of Health, Department of Health and Human Services, Research Triangle Park, North Carolina, United States of America; Stony Brook University, Graduate Program in Public Health, United States of America

## Abstract

Inorganic arsenic (iAs) is an established transplacental agent known to affect fetal development in animal studies. However, iAs has not been adequately studied in the general population with respect to iAs exposure during pregnancy and its impact on the health status of newborns. The aims of this study were to 1) elucidate the association between arsenic exposure and oxidative/methylated DNA damage in pregnant women, and 2) determine the association with birth outcomes. A birth cohort study of 299 pregnant mother-newborn pairs was recruited during 2001–2002 in Taiwan. We collected maternal urine samples during the 3^rd^ trimester for measuring iAs and its metabolites. We used high-performance liquid chromatography/inductively coupled plasma mass spectrometry (HPLC-ICP-MS) for quantifications of the arsenic species. Liquid chromatography/tandem mass spectrometer (LC-MS/MS) was used to measure the 8-oxo-7,8-dihydro-2′-deoxyguanosine (8-oxodG) and N^7^-methylguanosine (N^7^-MeG) DNA damage biomarkers. Birth outcomes were collected to assess the associations with maternal arsenic exposure and the DNA damage biomarkers. Multiple regression analyses showed that maternal urinary iAs had positive associations with the methylated N^7^-MeG (beta  = 0.35, *p*<0.001) and oxidative 8-oxodG (beta  = 0.24, *p*<0.001) DNA damage biomarkers, and a decreased one-minute (1-min) Apgar score (beta  = -0.23, *p* = 0.041). Maternal N^7^-MeG was also associated with a decreased 1-min Apgar score (beta  = −0.25, *p* = 0.042). Mutual adjustment for iAs and N^7^-MeG showed an independent and significant prediction for a decreased 1-min Apgar score of iAs (beta  = −0.28, *p* = 0.036). Maternal iAs exposure was associated with both maternal DNA damage and adverse newborn health. Maternal N^7^-MeG levels might be a novel biomarker for monitoring fetal health related to iAs.

## Introduction

Health status at birth is an important determinant of morbidity and mortality in early childhood [1] and of chronic disease in adulthood [2]. For example, birth weight is not only reflective of maternal health status, but also predictive of the probability for newborn survival, development, and long-term health [3]. The Apgar score is a routine for evaluating the physical condition of the newborn, including heart rate, respiratory effort, muscle tone, reflex irritability, and skin color shortly after delivery. A score ≥7 indicates that the condition of the newborn is good-to-excellent [4]. Otherwise, immediate extra medical care or even an intensive care unit admission would be necessary. Newborns with low birth weight (LBW) or low Apgar scores often develop various negative health consequences. Long-term effects of LBW include increased risk of cardiovascular disease, type 2 diabetes mellitus, and impaired reproductive function [5]. Several studies have shown that low Apgar scores may be associated with an increased risk of reduced cognitive function and increased learning difficulties later in life [6]. Arsenic is a well-known toxicant and carcinogen, and increasing evidence indicates that arsenic may adversely affect pregnancy outcomes and development of the newborn.

Arsenic easily crosses the placenta [7], and even moderate exposure to arsenic during pregnancy has been associated with adverse health outcomes in the fetus [8]. Studies have shown that prenatal arsenic exposure is inversely associated with birth weight in Bangladesh [9], [10], and Inner Mongolia [11]. Prenatal arsenic exposure at low-to-moderate levels might also have effects on the fetus, but more evidence is needed [12].

Disrupted placentation [13] and endocrine disturbance [14] have been reported for arsenic-related adverse pregnancy outcomes. However, the mechanisms require further investigation. Studies of mice have reported that arsenic leads to an increase in oxidative stress and a subsequent increase in 8-oxo-7,8-dihydro-2′-deoxyguanosine (8-oxodG) in tissues of tested animals [15], [16]. Increased urinary 8-oxodG in pregnant women has been linked to arsenic exposure [17]. Recently, maternal DNA damage mediated by arsenic exposure has been proposed as one mechanism responsible for fetal programming [18]. There is, however, little information on the formation of methylated DNA damage induced by arsenic exposure. Arsenic-treated mice have an arsenic-related increase in hepatic N^7^-methylguanine (N^7^-MeG), a marker of methylated DNA damage that reflects the overall rate of DNA methylation [19], [20], but this finding has not been confirmed in humans.

We sought to determine whether prenatal exposure to low-to-moderate levels of arsenic is associated with maternal oxidative/methylated DNA damage, and to evaluate the associations with birth outcomes. We found decreased Apgar score was associated prenatal arsenic exposure, and the maternal methylated DNA damage as the biomarker in a general population from central Taiwan.

## Methods

### Ethics statement

The Human Ethical Committee of the National Health Research Institutes in Taiwan approved this study. Before participating in the study, all pregnant women signed informed consent forms after receiving detailed explanations of the benefits and risks.

### Study participants

**Figure 1:**
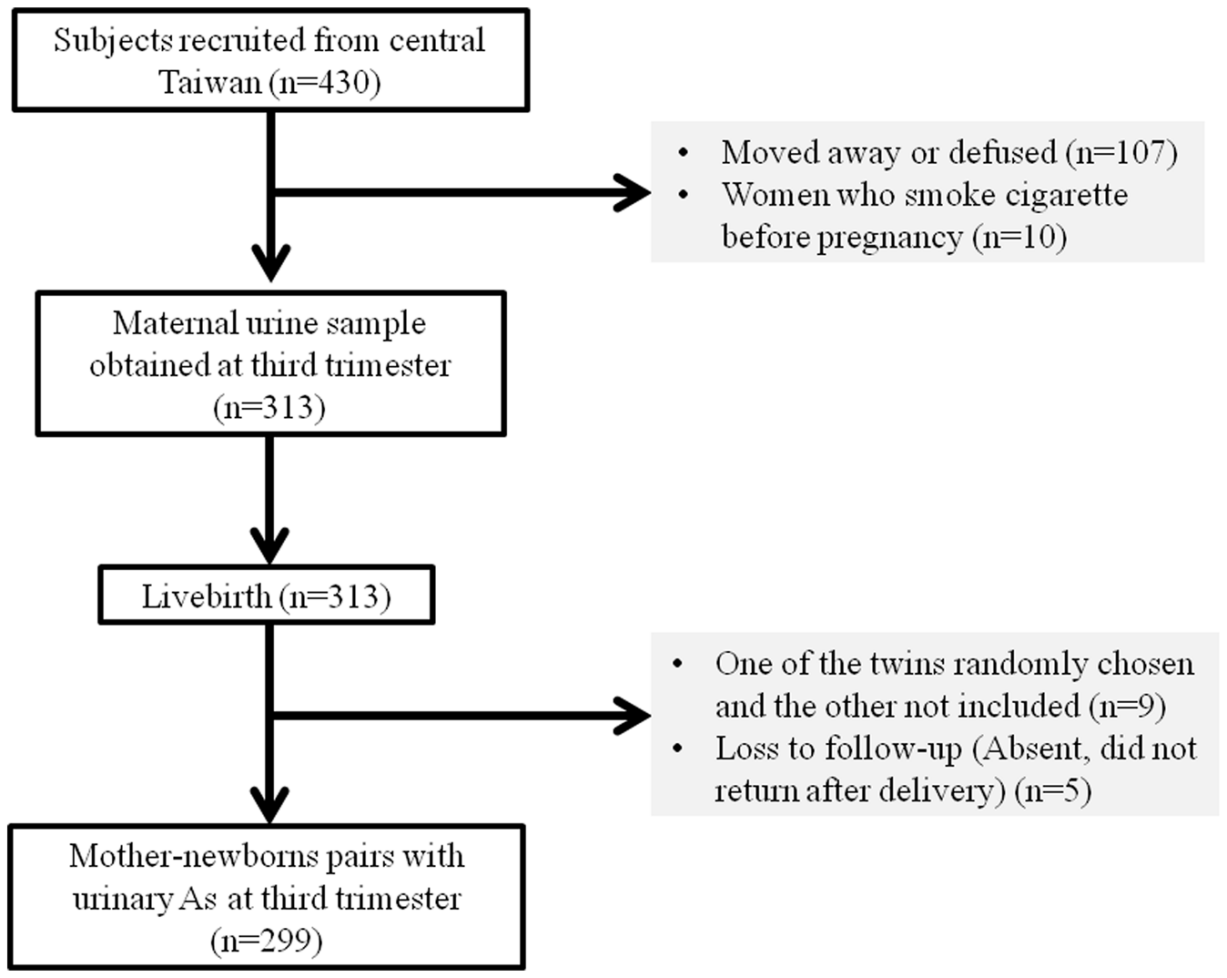
Subject recruitment and follow-up scheme for final analyses.

There is no record of high arsenic exposure in this study region; and, a representative sample for the general population was targeted. Subject recruitment [21] and preliminary results [22] were described previously. Briefly, all pregnant women who received care at the local medical center serving the general population were invited to join our study between December 2000 and November 2001 (Figure 1). The pregnant women were generally healthy and living in the local area with good prenatal appointment compliance. Initially, 430 of 610 pregnant women were volunteers recruited at 8 weeks gestation on average. The subjects provided urine samples during the third trimester (28–38 weeks gestation). Of the 430 pregnant women, 107 did not have sufficient data or provide urine, and 10 women who smoked cigarettes were excluded. To facilitate compliance with an independent outcomes assumption, one of the two subjects was randomly selected for the nine pairs of twins. In addition, we had missing data on 5 newborn heath assessments due to loss to follow-up. Consequently, a total of 299 mother-newborn pairs were reported.

### Determination of arsenic metabolites

The pregnant women collected urine in a 200-ml paper cup. The research assistant then allocated the urine specimens to 10-ml polypropylene tubes and stored them in a −20°C freezer until analysis. The concentrations of arsenite (As^3+^), arsenate (As^5+^), monomethylarsonic acid (MMA), and dimethyl arsenate (DMA) in the urine samples were measured using high-performance liquid chromatography/inductively coupled plasma mass spectrometry (HPLC-ICP-MS). We used anion exchange columns (Hamilton PRP X-100 [10 µm particle size, 250 mm×4.1 mm]) for arsenic speciation. The mobile phase was 100 mmol/ L Na_2_HPO_4_/ NaH_2_PO_4_ (pH = 5.75) at a flow rate of 1 mL/min. The limitations of detection (LOD) for the various species were 0.09 µg/L for As^3+^, 0.05 µg/L for As^5+^, 0.05 µg/L for MMA, and 0.04 µg/L for DMA.

Blanks were analyzed every 10 samples and the results were <0.01 µg/L of arsenic. Five standards spanning the range of urinary arsenic concentrations were used to set the calibration curve in each run of 10 samples. The analysis of samples proceeded only when the correlation coefficients of the standard curves with outcomes were >0.999. Duplicates were run every 10 samples and the coefficients of variance (CV %) were <5%. The recovery rate was between 90% and 110% with standard addition relevant to samples ranging from 2–5 µg/L for all arsenic species. The interbatch CVs were <10% in all samples ranging from 4.9%–7.8%. The inter-lab comparisons were conducted by participating in the Round Table Program organized by Recipe Chemicals and Instruments GmbH (Munich, Germany). We fulfilled the reference values for all tested samples.

Creatinine was measured by the Beckman Synchron LX20 auto-system (Beckman Coulter, Brea, CA, USA) in the central lab of Chung-Ho Memorial Hospital of Kaohsiung Medical University using a spectrophotometric method with picric acid as the reactive at 520 nm. We used total arsenic (tAs) as the sum of inorganic arsenic (As^3+^ and As^5+^) and organic arsenic (MMA and DMA) divided by the urinary creatinine in the subsequent analysis [23].

### DNA damage biomarker quantification

Urinary 8-oxodG and N^7^-MeG were determined using liquid chromatography/tandem mass spectrometry (LC-MS/MS). Urine samples were thawed at room temperature and centrifuged at 10000 g for 5 min. Then, 20 µL samples of urine were diluted 20 times with 96% acetonitrile containing 0.1% formic acid (FA; 380 µL). An 80-µL aliquot of diluted urine was spiked with 20 µL of a mixed solution consisting of ^15^N_5_-N7-MeG and ^15^N_5_-8-oxodG as an internal standard and then vortexed. A 100-µL sample of prepared urine was injected directly into the on-line solid-phase extraction column (Sep-Pak C18 cartridges, 1 g/6 mL; Waters, Milford, MA, USA). After automatic sample cleanup, LC-MS/MS analysis was done using a PE Series 200 HPLC system interfaced with a PE Sciex API 3000 triple quadruple mass spectrometer with an electrospray ion source [24] (Applied Biosystems, Toronto, Canada). The LOD of the 8-oxodG and N^7^-MeG was 0.01 µg/L and the coefficient of variation (CV) was 2.5% and 5.5% for the 8-oxo-dG and N^7^-MeG, respectively.

### Birth outcomes and covariates

The physical parameters of the newborns, including gestational age, sex, birth weight and height, head and chest circumference, and Apgar score, were measured and recorded by the same pediatrician and a well-trained assistant. The nurses in the maternity ward measured birth weight using a scale for babies (Misaki baby scale, Japan) that was calibrated before each use. Calibration with a standard weight (5 kg) at regular intervals showed that all scales were stable and precise. Birth length was measured to the nearest 0.1 cm using a wooden length board, and the head and chest circumferences were measured using a metal measuring tape. The Apgar score was determined for each baby to ascertain physical condition and determine the immediate need for extra medical or emergency care. A pediatrician or trained nurse assigned Apgar scores based on breathing effort, heart rate, muscle tone, reflexes, and skin color. Each category was scored with a 0, 1, or 2, according to the observed conditions. The Apgar test was given to babies at 1 and 5 minutes [25].

In the current study, adverse birth outcomes in newborns involved a low birth weight and low Apgar scores. We suggest that birth length might be correlated with the physical condition of the baby, such as body relaxation, and head and chest girth with the mode of delivery (i.e., normal spontaneous delivery, cesarean). In the present study, adverse birth outcomes in newborns were based on LBW and low Apgar scores. LBW newborns were defined as newborns with a birth weight <2,500 g. Newborns with low Apgar scores had a 1- or 5-min Apgar score <7 [4]. Information on demographic or socioeconomic factors, or other factors that confounded the associations between maternal arsenic exposure, maternal DNA damage, and newborn birth outcomes was collected. We used an administrative questionnaire to obtain the demographic data of the pregnant women, such as age, parity, education, and health-related data (medical history, as well as cigarette smoking and alcohol use before and after pregnancy).

### Statistical analysis

We assessed the associations between maternal arsenic exposure (iAs, MMA, and DMA), maternal DNA damage (8-oxodG and N^7^-MeG), and newborn health parameters (birth weight, birth length, head circumference, chest girth, and Apgar scores). Arsenic metabolites and DNA damage biomarker levels under LOD were recorded as one-half of the LOD values. All variables were assessed for normal distribution or natural logarithmic-transformation to approximate a normal distribution. Specifically, all urinary arsenic species levels were log-transformed, and the urinary levels of 8-oxodG and N^7^-MeG were natural log-transformed to obtain a normal distribution. Covariates assessed included maternal age at delivery, mode of delivery, pre-pregnancy BMI, gestational age, newborn sex, prenatal alcohol consumption, and maternal education.

To assess differences in the characteristics between subjects who were followed and subjects lost to follow-up, we used an independent sample *t*-test for continuous variables and the χ^2^ method for categorical variables (see Supplemental Material, Table S1). Pearson correlations were used to explore the associations between maternal arsenic levels, DNA damage, newborn health status, and other covariates. Significantly correlated variables were further analyzed in multivariable regression models adjusting for potential confounders. Potential confounders were identified based on correlations (*p*<0.1) with arsenic exposure and birth outcomes. There were no significant differences in the relationships in the birth outcomes and maternal iAs between the sexes; we combined data from female and male newborns to increase the statistical power.

Maternal age, pre-pregnant BMI, gestational age, mode of delivery, and newborn sex were included in the final model. We used birth outcomes as both continuous and binary variables in the linear regression model and the Cox's proportional hazards model, respectively. Statistical significance was set at a *p*<0.05. For the Cox's proportional hazards model estimates, we transformed continuous variables into a two-level scale to compare the above and below medians of the arsenic metabolites and DNA damage biomarkers. We calculated the 95% CIs of the relative risks from the corresponding regression coefficients and standard errors. All statistical analyses were performed using SPSS 18.0 software (SPSS, Inc., Chicago, IL, USA).

## Results

### General characteristics of the subjects

Table 1 presents the characteristics of pregnant women and the newborns by sex. Of the 299 newborns, 151 (51%) were male and 148 (49%) were female. Female newborns had significantly lower birth weights than the male newborns (3170 vs. 2985 gm, *p* = 0.001). Twenty-one (7%) newborns had a LBW (<2,500 g), 13 (61.9%) of which were female. Sixty-nine (23%) newborns had 1-min Apgar scores <7, of which 40 were male (*p* = 0.15 for sex difference). In addition, there were 4 female newborns with low 5-min Apgar scores as compared with none in males. Only 5 (1.7%) of women consumed alcohol during pregnancy. The characteristics for the excluded twins were similar to the included twins.

**Table 1:** Characteristics of mothers and their newborns by newborn sex in central Taiwan during 2000–2001 (n = 299).

Characteristics	All (n = 299)^a^	Male (n = 151)^a^	Female (n = 148)^a^	*p*-value^b^
*Pregnant women*				
Age (years)	28.3±4.2	28.1±4.0	28.0±4.8	0.772
BMI (Kg/m^2^)	25.6±3.9	25.6±4.4	25.1±4.0	0.832
Urinary creatinine (mg/dL)	78.2±48.5	74.9±52.3	80.7±46.6	0.732
Parity				0.693
Primiparous	159 (53)	90 (59)	69 (47)	
Multiparous	140 (47)	61 (41)	79 (53)	
Maternal Education				0.482
≤ high school	132 (44)	61 (41)	71 (48)	
high school +2years	117 (39)	60 (40)	57 (39)	
≥ high school +3 years	50 (17)	30 (19)	20 (13)	
Alcohol consumption				0.783
Yes	5 (1)	2 (1)	3 (1)	
No	294 (99)	149 (99)	145 (99)	
*Newborns*				
Gestational age (weeks)	39±2.8	39±1.5	39±1.8	0.290
Birth length (cm)	51±2.6	51.5±2.3	50.7±2.7	**0.005**
Head circumference (cm)	34.5±18.5	35.6±25.1	33.2±1.5	0.261
Chest girth (cm)	32.7±1.8	32.9±1.8	32.5±1.8	0.120
Mode of delivery				
Normal spontaneous	99 (33)	45 (30)	44 (30)	0.534
Vacuum extraction	103 (34)	55 (36)	58 (39)	0.356
Cesarean section	97 (33)	51 (34)	46 (31)	0.289
Birth weight (g)	3085±466	3170±425	2985±493	**0.001**
<2,500	21 (7)	8 (5)	13 (9)	**0.019**
≥2,500	278 (93)	143 (95)	135 (91)	
One-minute Apgar score	8.3±1.0	8.2±0.91	8.3±1.15	0.664
<7	69 (23)	40 (26)	29 (20)	0.153
≥7	230 (77)	111 (74)	119 (80)	
Five-minute Apgar score	9.8±0.8	9.6±0.5	9.7±1.1	0.546
<7	4 (1)	0 (0)	4 (3)	**0.001**
≥7	295 (99)	151 (100)	144 (97)	

a Presented as the mean ± SD or number (percentage).

b *p*-value for difference between male and female newborns using *t*-test for continuous variables and χ^2^ or Fisher's exact test for categorical variables. The *p*-value was bolded when <0.1.

Women lost to follow-up had a significantly lower average gestational age and greater income than the women who were followed. In addition, the mothers followed tended to be older and had slightly lower pre-pregnant BMIs than the mothers lost to follow-up (*p*<0.1). No significant differences existed in parity, newborn birth outcomes, arsenic levels, mode of delivery, or maternal education and life styles between the groups (see Supplemental Material Table S1).

### Maternal arsenic exposure and DNA damage

The levels and distribution of arsenic metabolites in maternal urine are shown in Table 2. The median (5th–95th) levels of creatinine-adjusted urinary As^3+^, As^5+^, MMA, DMA, iAS, and tAS were 0.30 (0.06–1.77), 0.21 (0.05–2.95), 0.46 (0.07–5.82), 20.01 (1.43–69.94), 0.79 (0.18–3.96), and 22.26 (2.80–83.0) µg g cre^−1^, respectively. Among all arsenic metabolites, the frequency of samples with arsenic metabolite levels above the LODs was >92%. The median (5th–95th) concentrations of creatinine-adjusted urinary 8-oxodG and N^7^-MeG were 3.48 (1.59–8.56) µg g^−1^ cre and 12.88 (6.47–25.57) mg g^−1^, respectively (Table 2).

**Table 2:** Distribution of creatinine-adjusted concentrations of urinary arsenic species (iAs, MMA, and DMA) and urinary DNA damage biomarkers (8-oxodG and N^7^-MeG) for pregnant women in the present study versus two others with established arsenic-related effects (n = 299).

	< LOD	Percentile^b^						
Exposure variables^a^	(n)	Min	5^th^	25^th^	50^th^	75^th^	95^th^	Max
***As metabolites (µg g cre^−1^)***								
As^3+^	14	0.02	0.06	0.16	0.30	0.66	1.77	12.17
As^5+^	20	0.02	0.05	0.11	0.21	0.49	2.95	16.52
MMA	10	0.05	0.07	0.18	0.46	2.03	5.82	43.89
DMA	12	0.12	1.43	7.48	20.01	32.30	69.94	131.5
iAs (ΣAs^3+^, As^5+^)	-	0.05	0.18	0.38	0.79	1.49	3.96	18.00
tAs (ΣiAs, MMA and DMA)	-	0.21	2.80	8.21	22.26	36.72	83.00	137.5
***DNA damage biomarkers***								
8-oxodG (µg g cre^−1^)	0	0.24	1.59	2.67	3.48	4.84	8.56	16.94
N^7^-MeG (mg g cre^−1^)	0	0.21	6.47	10.28	12.88	19.50	27.57	76.49

a Abbreviations: iAs represents the sum of As^3+^ and As^5+^; MMA: methylarsonic acid; DMA: dimethylarsinic acid; AsB: arsenobetaine; tAs: the sum of iAs, MMA, and DMA; 8-oxodG: 8-oxo-7,8-dihydro-2′-deoxyguanosine; N^7^-MeG: N7-methylguanine.

b This study. Taiwanese pregnant women in third trimester (n = 299).

### Maternal arsenic exposure, oxidative/methylated DNA damage, and birth outcomes

Table 3 shows a significant positive correlation between maternal 8-oxodG levels and iAs (r = 0.24, *p*<0.001), MMA (r = 0.16, *p*<0.001), DMA (r = 0.13, *p*<0.05), and tAs (r = 0.17, *p*<0.001). Similarly, a significant positive correlation was shown between the maternal N^7^-MeG levels and iAs (r = 0.35, *p*<0.001) and the metabolites. In addition, a significant positive correlation was shown between maternal 8-oxodG levels and N^7^-MeG (r = 0.62, *p<*0.001). A negative correlation of −0.13 (p = 0.08) was found between maternal iAs levels and the 1-min Apgar scores of newborns. A significant positive correlation was noted between the maternal MMA levels and the 1-min Apgar scores of newborns (r = 0.15, *p* = 0.03). A suggested negative correlation might exist between the maternal N^7^-MeG levels and the 1-min Apgar scores of newborns (r = −0.18, *p* = 0.06). The maternal MMA and DMA levels were not significantly correlated with the birth outcomes of birth weight, length, and head and chest circumferences.

**Table 3:** Pearson correlations between maternal inorganic arsenic and its metabolites levels, DNA damage in pregnant women, newborn health status, and other factors.

Health parameter	Maternal urinaryAs species(µg g cre^−1^)^a^				Maternal urinary DNA damages (µg g cre^−1^)^c^	
	iAs^b^	MMA^b^	DMA^b^	tAs^b^	8-oxodG	N^7^-MeG
***Maternal urinary DNA damage*** c						
8-OHdG	0.24^**^	0.16^**^	0.13^*^	0.17^**^	1.00	
N^7^-MeG	0.35^**^	0.19^**^	0.23^**^	0.27^**^	0.62^**^	1.00
***Pregnant women***						
Maternal age (years)	0.06	0.07	**0.13^#^**	**0.12^#^**	0.16^*^	0.04
Pre-pregnant BMI (Kg/m^2^)	0.05	−0.02	**0.12^#^**	0.15^*^	−0.05	−0.01
Maternal education	−0.03	−0.09	−0.05	−0.09	−0.06	−0.06
***Newborns***						
Sex	0.04	−0.08	0.04	0.06	−0.05	−0.04
Gestational age (weeks)	−0.04	0.06	0.07	0.06	0.07	−0.02
Mode of delivery	0.08	−0.07	−0.06	−0.05	−0.08	−0.12
Birth weight (g)	0.03	0.13	0.09	0.12	0.04	0.09
Birth length (cm)	−0.03	0.09	0.03	0.05	−0.001	−0.01
Head circumference (cm)	0.09	0.01	0.02	0.05	0.02	−0.08
Chest girth (cm)	0.04	0.10	0.05	0.07	0.04	0.11
One-minute Apgar score	**−0.13^#^**	0.15^*^	0.11	0.10	0.06	**−0.18^#^**
Five-minute Apgar score	−0.01	0.07	0.10	0.09	0.01	0.06

a All urinary arsenic species in pregnant women were adjusted by creatinine and log-transformed.

b Pearson correlation coefficient; ^#^ *p*<0.10; *p<0.05; **p<0.01.The *p*-value was bolded when <0.1.

c Urinary 8-oxodG and N^7^-MeG in pregnant women were both adjusted by creatinine and natural log-transformed.

Regression analyses identified positive associations between the maternal urinary level of iAs, MMA tAs, and the DNA damage biomarkers 8-oxodG and N7-MeG (Table 4). Maternal iAs levels had negative associations with 1-min Apgar scores (β-coefficient  = −0.23, 95% CI: −0.29 − −0.18, *p* = 0.041). MMA had positive associations with the newborn birth weight (β = 0.25, 95% CI: 0.02–0.42, *p* = 0.034), and a marginally significant positive association with 1-min Apgar scores (β = 0.21, 95% CI: −0.01–0.44, *p* = 0.06). In addition, the DMA and tAs were not significantly associated with the 1-min Apgar score. The maternal urinary N^7^-MeG levels had negative associations with the birth length (β = −0.21, 95% CI: −0.86 − −0.13, *p = *0.024) and 1-min Apgar score (β = −0.25, 95% CI: −0.63 − −0.15, *p = *0.042). To determine whether or not the estimated effects of maternal iAs exposure on newborn health status was via methylated DNA damage, we performed a multivariable adjusted regression analysis to estimate the association between maternal iAs exposure and newborn health outcomes, controlling for N^7^-MeG and potential confounders (Table 5). The associations between maternal iAs levels and 1-min Apgar scores remained significant after adjusting for N7-MeG.

**Table 4:** Multiple linear regression (β-coefficient) for the association^a^ between birth outcomes with maternal urinary arsenic metabolites^b^ and DNA damage^c^ biomarkers (n = 299).

Health parameter	Maternal urinaryAs species (µg g cre^−1^)				Maternal urinaryDNA damage (µg g cre^−1^)	
	iAs^b^	MMA^b^	DMA^b^	tAs^b^	8-oxodG^c^	N^7^-MeG^c^
***Pregnant women***						
8-oxodG^c^						
β	**0.24**	**0.16**	0.08	**0.13**	-	**0.64**
95% CI	**0.16–0.43**	**0.05–0.26**	−0.02–0.17	**0.01–0.25**	-	**0.56–0.74**
*p*-value	**<0.001**	**0.03**	0.123	**0.028**	-	**<0.001**
N^7^-MeG^c^						
β	**0.35**	**0.17**	**0.19**	**0.27**	**0.62**	-
95% CI	**0.28–0.55**	**0.07–0.27**	**0.09–0.28**	**0.15–0.39**	**0.53–0.71**	-
*p*-value	**<0.001**	**0.001**	**<0.001**	**<0.001**	**<0.001**	-
***Newborns***						
Birth weight (g)						
β	0.01	**0.25**	0.10	0.17	0.05	−0.16
95% CI	−0.28–0.34	**0.02–0.42**	−0.13–0.33	−0.12–0.45	−0.25–0.34	−0.35–0.35
*p*-value	0.845	**0.034**	0.396	0.312	0.745	0.062
Birth length (cm)						
β	−0.19	0.398	0.06	0.18	0.04	**−0.21**
95% CI	−0.99–0.62	−0.15–0.95	−0.54–0.65	−0.54–0.90	−0.25–0.31	**−0.86 – −0.13**
*p*-value	0.648	0.157	0.854	0.626	0.886	**0.024**
Head circumference (cm)						
β	0.29	0.01	−0.07	−0.05	−0.09	−0.02
95% CI	−0.22–0.81	−0.35–0.35	−0.45–0.32	−0.47–0.46	−0.59–0.39	−0.81–0.76
*p*-value	0.259	0.970	0.750	0.985	0.696	0.053
Chest girth (cm)						
β	0.11	0.28	0.14	0.12	0.24	0.46
95% CI	−0.46–0.68	−1.22–0.69	−0.37–0.49	−0.41–0.64	−0.29–0.78	−0.11–1.02
*p*-value	0.699	0.169	0.798	0.655	0.376	0.116
One-minute Apgar score						
β	**−0.23**	**0.21**	0.19	0.24	0.13	**−0.25**
95% CI	**−0.29 – −0.18**	**−0.01–0.44**	−0.04–0.43	−0.05–0.52	−0.16–0.43	**−0.63 – −0.15**
*p*-value	**0.041**	**0.060**	0.104	0.102	0.390	**0.042**
Five-minute Apgar score						
β	0.02	0.09	0.14	0.15	0.04	−0.09
95% CI	−0.19–0.24	−0.07–0.24	−0.02–0.30	−0.04–0.34	−0.16–0.25	−0.15–0.29
*p*-value	0.861	0.269	0.083	0.125	0.692	0.413

a Multiple linear regression model was adjusted for maternal age, pre-pregnant BMI, mode of delivery, gestational age, and newborn sex; β, regression coefficient; 95% CI, 95% confidence interval. The *p*-value was bolded when <0.1.

b All maternal urinary As species were adjusted by creatinine and log-transformed.

c Urinary 8-oxodG and N^7^-MeG in pregnant women were both adjusted by creatinine and natural log-transformed.

**Table 5:** Multiple linear associations (β-coefficient) of birth outcomes with maternal urinary inorganic concentrations and DNA damage biomarkers after further mutual adjustment for each other.

Birth outcomes	Urinary iAs and DNA damage biomarkers (µg g cre^−1^)	
	iAs^a^	N^7^-MeG^b^
Birth weight (g)		
β	−0.07	**−0.06**
95% CI	−0.40–0.27	**−0.07–0.56**
*p*-value	0.493	**0.079**
Birth length (cm)		
β	−0.27	**−0.11**
95% CI	−1.11–0.58	**−0.42 – −0.07**
*p*-value	0.530	**0.052**
Head circumference (cm)		
β	0.21	**−0.03**
95% CI	−0.35–0.77	**−0.02–0.04**
*p*-value	0.459	**0.065**
Chest girth (cm)		
β	−0.11	**0.29**
95% CI	−0.72–0.50	**−0.05–0.63**
*p*-value	0.726	**0.082**
One-minute Apgar score		
β	**−0.28**	**−0.05**
95% CI	**−0.38 – −0.15**	**−0.38–0.04**
*p*-value	**0.036**	**0.058**
Five-minute Apgar score		
β	0.02	−0.05
95% CI	−0.23–0.23	−0.16–0.09
* p*-value	0.909	0.482

a Multiple linear regression model was adjusted for maternal age, pre-pregnant BMI, mode of delivery, gestational age, and newborn sex, and further adjusted for N^7^-MeG. The *p*-value was bolded when <0.1.

b Multiple linear regression model was adjusted for maternal age, pre-pregnant BMI, gestational age, and newborn sex, and further adjusted for iAs.

Cox's proportional hazard model demonstrated that the maternal iAs level over the median was associated with the risk of decreased Apgar scores (RR = 1.14, 95% CI: 1.03–2.32, *p* = 0.012; Table 6). The relative risks for decreased Apgar scores was significantly increased (OR = 1.88, 95% CI: 1.07–2.15, *p* = 0.03) for pregnant women with urinary N^7^-MeG levels over the median. Relative risks for decreased Apgar scores associated with maternal N^7^-MeG levels were 1.92 (95% CI: 0.98–2.01, *p* = 0.07) after adjusting for iAs and other potential confounders.

**Table 6:** Relative risks (RR)^a^ and 95% confidence interval (95% CI) for low birth weight and decreased one-minute Apgar score in relation to maternal urinary arsenic and DNA damage biomarker levels (n = 299).

Relativerisk	Low birth weight (<2500 g)			Decreased Apgar score (<7)		
	n	RR (95% CI)	*p*-value^d^	n	RR (95% CI)	*p*-value^d^
**RR** b						
***Arsenic metabolites (µg g^−1^ cre)***						
iAs						
>0.38	11	1.04 (0.68–1.59)	0.522	**37**	**1.14 (1.03–2.32)**	0.012*
≤0.38	10	1.0		32	1.0	
MMA						
>0.46	8	0.56 (0.23–1.31)	0.131	26	0.59 (0.28–1.17)	0.156
≤0.46	13	1.0		44	1.0	
DMA						
>20.01	7	0.55 (0.24–1.30)	0.124	22	**0.46 (0.22–0.95)**	0.035^*^
≤20.01	14	1.0		47	1.0	
***DNA damage biomarkers (µg g^−1^ cre)***						
8-oxodG						
>3.48	12	1.31 (0.54–3.13)	0.355	32	0.84 (0.41–1.74)	0.713
≤3.48	9	1.0		38	1.0	
N^7^-MeG						
>12.88	13	**1.59 (0.88–2.86)**	**0.060^#^**	45	**1.88 (1.07–2.15)**	0.030*
≤12.88	8	1.0		24	1.0	
**ARR** c						
*** iAs***						
>0.38	11	1.02 (0.66–2.26)	0.625	**37**	**1.21 (1.10–2.45)**	0.006*
≤0.38	10	1		32	1	
*** N^7^-MeG***						
>12.88	13	1.49 (0.89–1.96)	0.342	45	**1.92 (0.98–2.01)**	**0.07^#^**
≤12.88	8	1		24	1	

a The continuous variables were transformed into a two-level scale using medians, which represented the high or low levels arsenic/DNA damage biomarkers exposure, to calculate relative risks (RR).

b Adjusted for potential confounders (maternal age, mode of delivery, pre-pregnant BMI, gestational age, and newborn sex).

c Adjusted for iAs or N^7^-MeG and potential confounders.

d Cox's proportional hazards model:^ #^ *p*<0.10; *p<0.05 for RR significance above 1. *p*-value was bolded when less than 0.1.

## Discussion

This is the first study to simultaneously assess the effects from maternal arsenic exposure and maternal oxidative and methylated DNA damage on the health status of newborns. The concentrations of urinary tAs in this study were significantly lower than previous studies conducted in Bangladesh (median urinary tAs, 717.5 µg g^−1^ cre) [26] and Chile (median urinary tAs, 40.4 µg g^−1^ cre) [27]. Adverse birth outcomes and decreased 1-min Apgar scores were associated with increased maternal levels of iAs and N^7^-MeG in the general population.

We observed a significantly increased relative risk of low Apgar scores associated with maternal urinary iAs levels after adjusting for N^7^-MeG. The critical mechanism underlying prenatal arsenic exposure is still not clear. Disrupted placentation [13] and endocrine disturbance [14] have been reported for arsenic-related adverse pregnancy outcomes. Animals treated with high doses of arsenic have reported severe early effects, such as neural tube effects [28]; however, the levels of arsenic exposure relevant to humans await further investigation. DNA damage was reported to be induced by iAs, as shown by increased concentrations of the biomarkers of oxidized DNA adducts (8-oxodG) in the brains of mice [29] and in the urine of women in the early stages of pregnancy [17]. In the present study, urinary 8-oxodG andN^7^-MeG levels were significantly associated with concentrations of maternal urinary arsenic species. N^7^-MeG was significantly associated with decreased birth length and 1-min Apgar scores after adjustment for maternal age, pre-pregnant BMI, mode of delivery, gestational age, and newborn sex. Thus, N^7^-MeG appeared to be a more sensitive biomarker than 8-oxodG for maternal DNA damage related to newborn adverse outcomes. This suggests that maternal iAs might cause both DNA damage and adverse newborn health independent of the DNA damage.

We first reported that the association between iAs and N^7^-MeG. N^7^-MeG has been established as a marker of human exposure to methylating agents and smoking [30]. The induction of N^7^-MeG is believed to occur via the direct toxicity of arsenic on enzyme activities, such as DNA repair enzymes. It has been shown that low concentrations of iAs are able to inhibit the expression and activity of poly(ADP-ribose) polymerase (PARP) enzymes [31], the enzymes responsible for base excision repair pathways, which increase unrepaired DNA lesions [32]. Urinary N^7^-MeG is derived from transfer RNA (tRNA) turnover [33]. The current association between arsenic exposure and increased urinary N^7^-MeG levels might be due to upregulation of alternative repair pathways of methylated DNA damage, such as base excision repair, resulting in the formation of N^7^-MeG. A previous report indicated that a variety of methylated purines and pyrimidines are found in tRNA, thus degradation of tRNA might result in the release of all minor methylated bases [34]. A recent *in vivo* study showed that arsenic exposure induces tRNA modification in *Saccharomyces cerevisiae*, and may subsequently increase cellular levels of N^7^-MeG [35]. An improper alteration in tRNA modification may lead to disorders in embryonic development, cell proliferation, and differentiation in mice [36], and developmental abnormalities in *Caenorhabditis elegans* [37].

DNA methylation is an important mechanism of fetal programming during fetal development [2], [10]. Long-term exposure to arsenic *in utero* has been associated with changes in DNA methylation, which may have severe consequences for the development of fetal health effects [10]. Urinary N^7^-MeG could be used as a biomarker for the alteration of DNA methylation due to arsenic [38]. It has been shown that high arsenic exposure in pregnant mice significantly reduces DNA methylation in offspring [39]. We suggest that iAs might induce adverse birth outcomes. Further evaluation is needed in a larger sample to understand whether or not prenatal low iAs exposure results in altering DNA methylation in newborns.

Maternal urinary DMA was not significantly associated with maternal 8-oxodG nor were 1-min Apgar scores. This might result from organic arsenic as a source of DMA and tAs: A large population study showed that seafood intake was a major determinant of increased urine concentrations of DMA and total arsenic [40]. Current study area residents in Taiwan regularly consume seafood and fish from the surrounded Pacific Ocean [41]. These foods might contain substantial amounts of arsenosugars or arsenolipids, which could metabolized as DMA [41].

The main strengths of this study include a study design that allows findings on the longitudinal capture of biomarkers for inorganic arsenic exposure to relate to birth outcomes, monitoring biomarkers of oxidative damage, and sufficient sample size. In addition, none of the study subjects smoked cigarettes, which otherwise would have been a major confounder because smoking is associated with several adverse outcomes of pregnancy, including LBW [42] and increased N^7^-MeG levels [30].

The current study was limited by the lack of paternal data, thus early contributions from the male complement due to arsenic-induced damage cannot be evaluated and warrant further studies. Furthermore, iAs has a short half-life and the efficiency of inorganic arsenic methylation to DMA was increasing since the 1^st^ trimester of pregnancy [43]. We only measured urinary arsenic species once during the third trimester, which likely introduced a substantial degree of exposure measurement misclassification towards null hypothesis.

In this study, there were no significant differences in the association of birth outcomes with maternal iAs between sexes; we combined data from female and male newborns to increase the statistical power. Future work might recruit more subjects to further verify the sex difference. We assessed arsenic and biomarkers of DNA damage using the same spot urine and thus temporality cannot be evaluated. However, we do not think the converse is likely to occur (i.e., greater oxidation/methylation metabolism increases arsenic elimination).

## Conclusions

This is the first study to report significant associations between arsenic-induced N^7^-MeG levels of pregnant women and an increased risk of adverse birth outcomes in newborns. These findings strongly emphasize that maternal N^7^-MeG may be a sensitive and effective biomarker for newborn health, particularly after early-life arsenic exposure. Further studies are necessary to understand the potential health effects of arsenic-related DNA methylation or tRNA modifications in newborns.

## Supporting Information

Table S1 **Demographic characteristic of followed and lost-to-follow-up subjects.**
(DOC)
