# Supplementary material for: Maternal Arsenic Exposure and DNA Damage Biomarkers, and the Associations with Birth Outcomes in a General Population from Taiwan
Source: PLoS One. 2014 Feb 18;9(2):e86398. doi: 10.1371/journal.pone.0086398 (PMC3928045; doi:10.1371/journal.pone.0086398)
Supplement: Table S1 — Demographic characteristic of followed and lost-to-follow-up subjects. (DOC) [file pone.0086398.s001.doc]

Supporting Information Legend: Table S1. Demographic characteristic of followed and lost-to-follow-up subjects

| Demographic characteristics | |  | Lost-to-follow-up (n=131) | |  | p-value^a^ compared to followed |
| --- | --- | --- | --- | --- | --- | --- |
| ***Continuous variables*** | |  | Mean (GM) | SD (95% CI) |  |
| Maternal age (years, n=203/126) | |  | 27.95 | 3.9 |  | **0.057** |
| Maternal BMI (n=199/121) | |  | 25.8 | 3.4 |  | **0.078** |
| Birth weight (gram, n=299/126) | |  | 3241 | 374 |  | 0.435 |
| Birth length (cm, n=299/126) | |  | 50.8 | 2.8 |  | 0.509 |
| Gestational age (weeks, n=299/126) | |  | 32.0 | 15.7 |  | **0.001** |
| Head circumference (cm, n=299/126) | |  | 33.3 | 1.6 |  | 0.855 |
| Chest circumference (cm, 299/125) | |  | 32.4 | 1.9 |  | 0.987 |
| One-min Apgar score (n=299/125)^c^ | |  | 8.4 | 0.5 |  | 0.357 |
| Five-min Apgar score (n=299/125)^c^ | |  | 9.8 | 0.4 |  | 0.497 |
| Maternal U-iAs (μg l^-1^, n=299/120) | |  | 0.7 (0.4) | 1.7 (0.2-1.8) |  | 0.306 |
| Maternal U-MMA (μg l^-1^, n=299/120) | |  | 1.2 (0.4) | 3.2 (0.1-5.9) |  | 0.265 |
| Maternal U-DMA (μg l^-1^, n=299/120) | |  | 16.3 (5.8) | 18.3 (0.1-55.8) |  | 0.410 |
| Maternal U-tAs (μg l^-1^, n=299/120) | |  | 18.3 (9.5) | 23.3 (0.5-62.0) |  | 0.423 |
|  | |  |  |  |  |  |
| **Categorical variables** | |  | n | % |  |  |
| Gender | Male |  | - | - |  | 0.850 |
|  | Female |  | - | - |  |  |
| Singleton | Yes |  | 130 | 99 |  | **0.095** |
|  | No (Twin^d^) |  | 1 | 1 |  |  |
|  | Missing |  |  |  |  |  |
| Mode of delivery |  |  |  |  |  |  |
|  | Normal spontaneous delivery |  | 29 | 22 |  | 0.421 |
|  | Vacuum extraction delivery |  | 43 | 33 |  | 0.552 |
|  | Cesarean section |  | 58 | 44 |  | 0.443 |
| Maternal education | ≤12 years |  | 58 | 53 |  | 0.672 |
|  | >12 years |  | 62 | 47 |  |  |
|  | Missing |  |  |  |  |  |
| Family annual income ($US/year) | <20,000 |  | 54 | 46 |  | **0.045** |
|  | ≥ 20,000 |  | 64 | 54 |  |  |
|  | Missing |  |  |  |  |  |
| Before pregnancy |  |  |  |  |  |  |
| Active smoker | Yes |  | 18 | 15 |  | 0.712 |
|  | No |  | 102 | 85 |  |  |
| Passive smoke | Yes |  | 53 | 45 |  | 0.796 |
|  | No |  | 66 | 55 |  |  |
| During pregnancy |  |  |  |  |  |  |
| Active smoker | Yes |  | 4 | 3 |  | 0.187 |
|  | No |  | 113 | 97 |  |  |
| Alcohol consumption | Yes |  | 5 | 4 |  | 0.268 |
|  | No |  | 114 | 96 |  |  |

^a^ Statistical method: Independent t-test, chi-square test. p-value was bolded when less than 0.1.

^b^ Number of followed and lost-to-follow-up subjects (n=follow/lost-followed up)

c Apgar score is the sum score of five vital sign, including appearance, pulse, grimace, activity, respiration, in newborns.

^d^ One of the twins was randomly chosen.
